# Designing a Collaborative Patient-Centered Digital Health Platform for Pediatric Diabetes Care in British Columbia: Formative Needs Assessment by Caregivers of Children and Youths Living With Type 1 Diabetes and Health Care Providers

**DOI:** 10.2196/46432

**Published:** 2023-07-13

**Authors:** Fatema S Abdulhussein, Susan Pinkney, Matthias Görges, Tibor van Rooij, Shazhan Amed

**Affiliations:** 1 Department of Pediatrics University of British Columbia Vancouver, BC Canada; 2 BC Children's Hospital Research Institute Vancouver, BC Canada; 3 Department of Anesthesiology Pharmacology & Therapeutics University of British Columbia Vancouver, BC Canada; 4 Department of Computer Science University of British Columbia Vancouver, BC Canada

**Keywords:** application design, challenge, child, design, development, diabetes, diabetic, digital health, digital solution, engagement, feature, needs assessment, patient engagement, patient need, pediatric, perception, privacy, secure, security, trust, Type 1 diabetes, Type 1, usage, user centered, user need, youth

## Abstract

**Background:**

Digital health apps are becoming increasingly available for people living with diabetes, yet data silos continue to exist. This requires health care providers (HCPs) and patients to use multiple digital platforms to access health data.

**Objective:**

In this study, we gathered the perspectives of caregivers of children and youths living with type 1 diabetes (T1D) and pediatric diabetes HCPs in the user-centered design of TrustSphere, a secure, single-point-of-access, integrative digital health platform.

**Methods:**

We distributed web-based surveys to caregivers of children and youths living with T1D and pediatric diabetes HCPs in British Columbia, Canada. Surveys were designed using ordinal scales and had free-text questions. Survey items assessed key challenges, perceptions about digital trust and security, and potential desirable features for a digital diabetes platform.

**Results:**

Similar challenges were identified between caregivers of children and youths living with T1D (n=99) and HCPs (n=49), including access to mental health support, integration of diabetes technology and device data, and the ability to collaborate on care plans with their diabetes team. Caregivers and HCPs identified potential features that directly addressed their challenges, such as more accessible diabetes data and diabetes care plans. Caregivers had more trust in sharing their child’s data digitally than HCPs. Most caregivers and HCPs stated that an integrative platform for T1D would support collaborative patient care.

**Conclusions:**

Caregiver and HCP perspectives gathered in this study will inform the early prototype of an integrative digital health platform. This prototype will be presented and iterated upon through a series of usability testing sessions with caregivers and HCPs to ensure the platform meets end users’ needs.

## Introduction

The COVID-19 pandemic has shown the promise of digital health to transform health care delivery and improve patient experience and clinical care outcomes through virtual care visits, mobile health, telemedicine, and remote patient monitoring [1]. An estimated 300,000 digital health apps are available, and more than 200 new apps are added daily [2]. Specifically, many stand-alone diabetes digital apps do not integrate regulated medical devices, such as insulin pumps or continuous glucose monitors (CGMs) [3,4]. However, the uptake of digital health apps has lagged as they are not user-friendly and difficult to navigate [5]. There are also concerns around digital health literacy, data privacy and security, and barriers to accessing technology for populations in varying socioeconomic and geographic backgrounds [1].

Most diabetes digital apps are designed for self-management, targeting patients living with type 2 diabetes [4,6]. In a systematic review that evaluated 656 diabetes health apps for iOS and Android in patients 50 years and older, it was found that 54% of the apps were limited to providing 1 function, which was a documentation feature [3]. This entailed manual recording of blood glucose values, monitoring of food, physical activity, and medication frequency, often linked with a reminder function [3]. However, only 18% of the diabetes apps allowed analysis of the recorded data and graphical results display [3]. A total of 96% of these apps were designed specifically for patients, but only 3.7% addressed the needs of both patients and health care providers (HCPs) [3].

User-centered design of digital health apps is an approach that incorporates stakeholder input at any point during the development process, from as early as when the idea is first conceived, as part of usability testing of initial prototypes that undergo iterative development, to the eventual pilot implementation of a minimal viable product [7,8]. User-centered design is considered necessary to ensure feasibility, usability, and eventual uptake of digital health solutions by end users, such as HCPs and patients [7]. User engagement in evaluating diabetes digital apps is low, and most apps have involved patients only after the apps have been created [6]. Some apps have studied the needs and key features desired by patients with diabetes in the design of digital health platforms [9-11]. Adults living with diabetes prioritize access to their diabetes data and communication with the health care team as key functions in diabetes apps [9,11]. A diabetes app that motivates adolescents with type 1 diabetes (T1D) to self-manage their diabetes by recording their glucometer data identified social interaction in a group, customization, and tangible rewards as motivating factors to use the app [10]. However, the perceptions of HCPs in the design of these apps were not evaluated [9,10].

Children with T1D have the most significant uptake of diabetes technology from CGM use to automated insulin delivery systems [12], which store a vast amount of patient-generated health data. Data from these devices are uploaded onto various proprietary digital platforms, and HCPs, patients, and their families must access these platforms using multiple usernames and passwords [13]. Tidepool, a cloud-based open-source digital platform that can integrate data from any diabetes device, was created to break down the siloed data [13]. Digital apps that provide education on diabetes self-management or in response to blood glucose data are limited but desired by patients [14]. Apps that allow people living with diabetes to interact with their health care team are limited and are mostly designed for adults living with type 2 diabetes [15,16].

To our knowledge, a digital platform that enables diabetes data integration from various devices while also providing easy access to diabetes care plans, educational resources, and a seamless digital connection to a diabetes health care team does not yet exist. As diabetes devices become increasingly interoperable, there is an unprecedented opportunity for automated streaming and integration of patient-generated health data into 1 trusted digital space viewable by both patients and their caregivers and HCPs, where they can also access information that will support the optimization and personalization of care experiences and diabetes self-management such as insulin dose adjustment.

Our transdisciplinary research team partnered with industry partners to assemble the TrustSphere consortium. This consortium aims to cocreate a digital health platform to support a collaborative and continuous care experience for patients, their caregivers, and their HCPs. TrustSphere provides a secure, single digital access point to view diabetes data and care plans. This study describes TrustSphere’s user-centered design approach at the conceptual phase of developing our digital health platform. The objective of this study was to gather perspectives of caregivers of children and youths living with T1D and pediatric diabetes HCPs on their current challenges, barriers, and facilitators of digital technology trust and usage for clinical care, and desired features early in the conceptual stage of designing this single-point-of-access secure integrative digital health platform.

## Methods

### Description of TrustSphere

At this early, formative stage of our project, the TrustSphere platform existed as a high-level concept and was described to caregivers of children and youths living with T1D as follows:

A secure online platform that will be customized for child and youth patients and their families, and will integrate a patient’s health information such as diagnoses, medications and treatments, appointments, lab test results, wearable data (eg, FitBit), etc. This platform would use secure and trusted digital identification and follow the highest health care industry and public standards of privacy protection. The platform would help make it easier for children and families to access their health information and care plans and to communicate directly with health care providers. It would also allow users to share their health information and care plans, if desired, with others involved in their child’s care, as well as donate their data confidentially for research.

TrustSphere was described to HCPs as follows:

… a patient-centered integrated digital platform customized for patients and families of children and youth with T1D, and for the health care providers who serve them. This integrated patient platform will use secure and trusted digital identification and will be in compliance with the highest health care industry and public standards of privacy protection. It will provide a single-point-of-access dashboard for care providers and patients that will integrate patient data from a range of sources including clinical data from EMRs and labs, glucose sensors and insulin pumps, and wearables (eg, FitBit), and will enable the creation of personalized patient care plans. This integrated platform will allow for direct communication between patients/families and members of their care team, and the ability for patients to share their medical data and diabetes care plans, if desired, with other individuals within their circle of care (ie, primary care, nursing support services, etc).

### Ethics Approval

This study was approved by the University of British Columbia Children’s and Women’s Research Ethics Boards (H20-03105).

### Data Collection

For caregivers of children and youths living with T1D in British Columbia (BC), a 35-item web-based survey was developed (Multimedia Appendix 1), which gathered perceptions of digital trust and security, challenges related to caring for their child with T1D, perspectives toward the proposed digital platform, importance of various potential features, and likelihood of future platform use based on the description above.

A 25-item web-based survey was developed for pediatric diabetes HCPs using REDCap (REDCap Consortium) (Multimedia Appendix 2) [17]. Survey items assessed critical current challenges in HCPs’ practice when providing care to children and youths with T1D, essential features in digital tools that support patient care, levels of concern around digital security and data privacy, and the likelihood of use of the digital platform based on the description above.

The surveys had a list of potential features that were compiled through a review of features in existing T1D platforms and apps and consultation with clinical stakeholders on the study team. Free text “other” answer categories were also offered to ensure respondents could describe features that may not be listed if needed.

### Participants

Using the BC Children’s Hospital (BCCH) Diabetes Clinic database, email invitations with study information and a survey link were distributed to caregivers of patients living with T1D who were younger than 18 years, who were accessing pediatric diabetes care at BCCH (n=760). A total of 99 caregivers of children and youths living with T1D in BC completed the survey with a response rate of 13%.

The HCP survey was distributed to pediatric diabetes HCPs (pediatricians, pediatric endocrinologists, diabetes educators, and dietitians) across the Canadian province of BC using the Annual BC Pediatric Diabetes Day email listserv (maintained by the University of British Columbia’s Continuing Professional Development administrative staff). This conference is attended by pediatric diabetes clinicians, such as physicians, diabetes nurse educators, dietitians, and pharmacists (n=232). The email contained information about the study and a link to the survey. A total of 49 HCPs completed the survey with a response rate of 21%.

### Analysis

Descriptive statistics were used to analyze quantitative survey data results. Qualitative data obtained through open-ended survey questions underwent conventional content analysis by a single coder trained in qualitative analysis to generate preliminary coding categories; 2 researchers then reviewed results independently, after which both researchers worked together to deliberate and finalize the coding guide, resolve inconsistencies, and identify themes.

## Results

### Participant Demographics and Technology Use

Table 1 shows the demographics of total caregiver respondents of children and youths living with T1D (n=99), though important to note that not all caregivers completed each section. The breakdown of HCP respondents (n=49), the Health Authority in which they practice within the province of BC, and the number of children and youths younger than 18 years with T1D followed by their practice are shown in Table 2.

Caregivers reported that 66% (65/99) of their children used CGM, and 54% (53/99) used an insulin pump. A total of 27% (27/99) reported that their children used health and well-being monitoring apps on their smartphones, while 34% (33/99) of caregivers used similar apps. Many caregivers (76/99, 77%) also stated that if their child was older than 7 years, they shared the decision-making responsibility when managing their diabetes.

**Table 1:** Participant characteristics of caregivers of children and youths who are <18 years old with type 1 diabetes (total caregiver respondents, N=99).

Characteristic		Participants
**Gender (n=94), n (%)**		
	Female	71 (76)
	Male	23 (24)
**Area of residence (n=94), n (%)**		
	Urban	42 (44)
	Suburban	40 (43)
	Rural	12 (13)
**Household income (CAD $^a^; n=85), n (%)**		
	Less than 45,000	6 (7)
	45,000 to less than 75,000	9 (11)
	75,000 to less than 100,000	7 (8)
	100,000 to 150,000	33 (39)
	150,000 to 300,000	21 (24)
	Greater than 300,000	9 (11)
**Education (n=91), n (%)**		
	High school graduation or less	5 (6)
	Graduated from trade school	3 (3)
	Some college or university degree	24 (26)
	College or university undergraduate degree	33 (36)
	College or university graduate degree	26 (29)

^a^CAD $1=US $0.76.

**Table 2:** Participant characteristics of health care providers (HCPs).

		Participants (n=49), n (%)
**HCPs**		
	Diabetes nurse educators	18 (37)
	Nursing support services^a^	12 (25)
	Pediatricians	9 (18)
	Dieticians	6 (12)
	Pediatric endocrinologists	4 (8)
**HCP health authority of practice**		
	Vancouver Island Health Authority	11 (22)
	Provincial Health Services Authority	8 (16)
	Northern Health Authority	8 (16)
	Fraser Health Authority	8 (16)
	Interior Health Authority	8 (16)
	Vancouver Coastal Health Authority	6 (12)
**Children and youths <18 years old with T1D^b^ in their practice**		
	Less than 25	20 (42)
	25-49	7 (14)
	50-99	6 (12)
	100-149	5 (10)
	150-200	3 (6)
	More than 200	8 (16)

^a^These health care providers support care of children living with type 1 diabetes in schools.

^b^T1D: type 1 diabetes.

### Reported Challenges

#### Caregivers of Children and Youths Living With T1D

Caregivers identified access to diabetes management technologies, diabetes support in schools, and mental health as the top challenges in caring for a child with T1D (Table 3). Qualitatively, they also identified barriers such as insufficient knowledge about diabetes technologies, access to financial coverage for diabetes technologies, and diabetes self-management skills (eg, insulin dose adjustments). Caregivers also reported that keeping track of all their child’s health information (55/99, 56%) and sharing this information among different HCPs (40/99, 40%) was challenging, with 65% (64/99) of caregivers using a combination of paper and digital sources.

**Table 3:** Challenges reported by caregivers of children and youths living with type 1 diabetes. (“What are the biggest challenges that you currently face related to caring for your child with T1D? Select your top 3.”)

Challenge	Participants (n=84), n (%)
Access to diabetes management technologies (ie. insulin pumps and glucose sensors)	45 (54)
Support for your child's diabetes care in school	45 (54)
Access to mental health support (social worker or psychologist)	41 (49)
Accessing your child's medical information (ie, glucose sensor data, pump data, and lab test results)	33 (39)
Connecting with your diabetes team between visits	32 (38)
Access to a pediatric diabetes doctor	17 (20)
Access to a registered dietician with experience in pediatric diabetes	9 (11)
Access to a diabetes nurse educator	5 (6)
Other	25 (30)

#### Diabetes HCPs

Table 4 outlines HCPs’ perspectives on the key challenges faced by patients and their families living with T1D, with the most common being related to access to mental health support (ie, a social worker or psychologist), diabetes management technologies (ie, insulin pumps and CGM), and their child’s clinical information. Qualitative data identified other challenges, including patients’ inability to implement HCPs’ recommendations into their day-to-day lives, financial barriers, and the different approaches to diabetes management at home versus at school.

**Figure 1:**
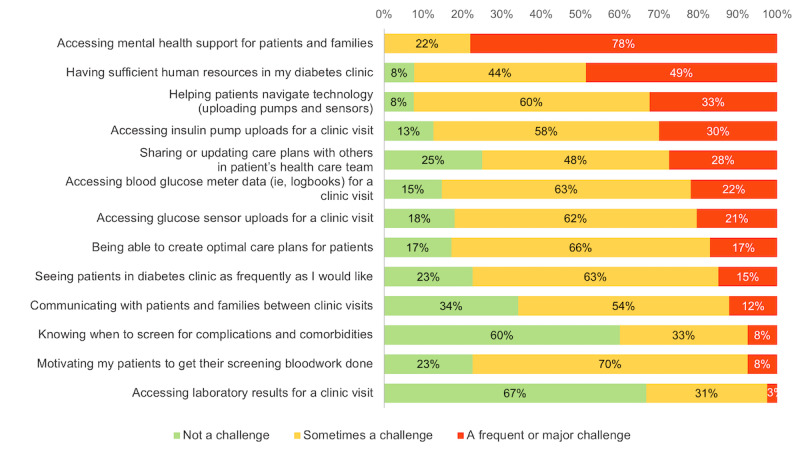
Current degree of challenges faced by health care provider in their practice. (“When providing care to children and youths <18 years of age with T1D in your clinic, to what degree do you find each of the following to be a current challenge in your practice? *Select one answer per row*.”).

HCPs reported similar challenges in their clinical care of children living with T1D. Insufficient mental health services (42/42, 100%), patients’ inability to use technology to prepare for a clinic visit (ie, uploading pump, and glucometer and CGM data; 37/41, 90%) leading to unavailability of insulin pump (35/41, 85%) and glucose data (35/42, 83%) during a clinical encounter, were identified as “frequent and major” or “sometimes” a challenge (Figure 1). Other challenges that were reported by HCPs qualitatively included a lack of web-based patient support resources (eg, guidance on filling out disability tax credit forms and paperwork for insurance companies), staying up to date on the latest diabetes technologies, having enough time to provide diabetes education during clinic visits, empowering patients to self-manage their diabetes between clinic visits, and lack of peer support programs.

**Table 4:** From the perspective of health care providers: current key challenges faced by patients with type 1 diabetes and their families. (“What are the current key challenges that your T1D patients and their families report facing? Select top 3.”)

Challenge	Participants (n=49), n (%)
Access to a pediatric diabetes doctor	6 (12)
Access to a diabetes nurse educator	5 (10)
Access to a registered dietitian with experience in pediatric diabetes	6 (12)
Access to mental health support (social worker or psychologist)	41 (84)
Access to diabetes management technologies (ie, insulin pumps and glucose sensors)	26 (53)
Support for their child’s diabetes care in school	17 (35)
Accessing their child’s clinical information (ie, glucose sensor data, pump data, and lab investigations)	19 (39)
Connecting with their diabetes team between visits	17 (35)
Other	5 (10)

### Preferences for Features of a Digital Health Platform for T1D

#### Caregivers of Children and Youths Living With T1D

**Figure 2:**
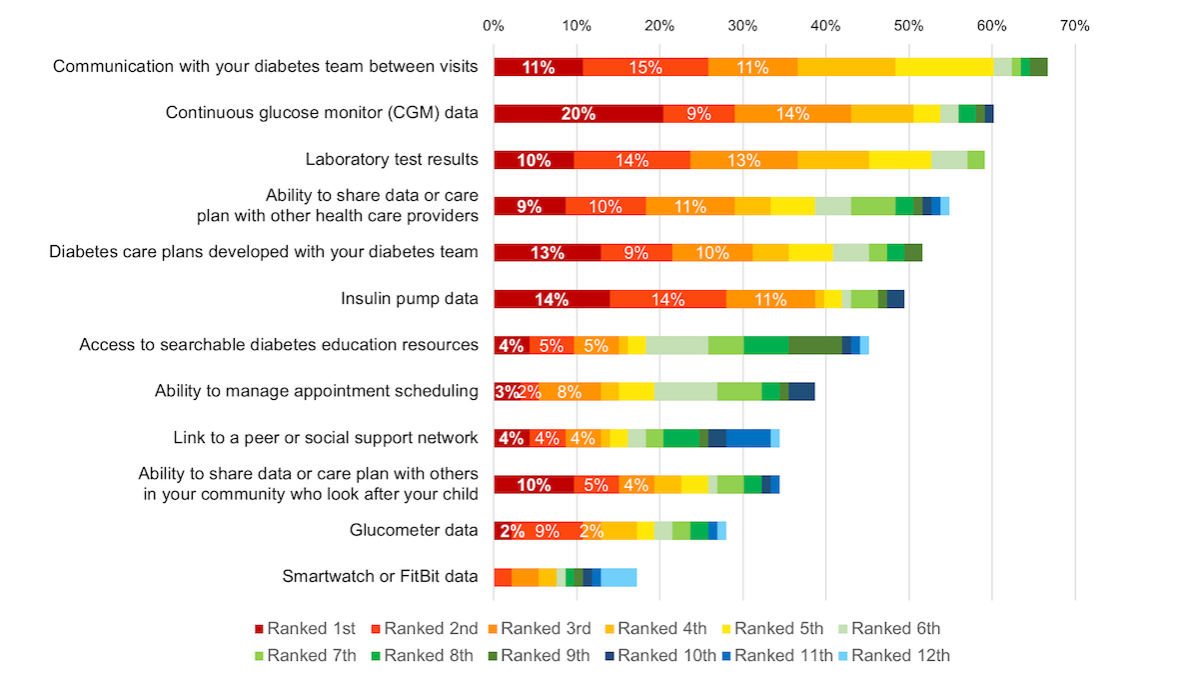
Priority ranking of information that caregivers of children and youths living with type 1 diabetes want to include in this digital platform (n=93). (“If this platform was customized for children and youths with type 1 diabetes and their parents/caregivers, what integrated data or features would be most useful for you? *Rank in priority*.”).

Caregivers were asked to rank the most useful features that could be offered by this platform. From the comprehensive list of potential features, 43% (40/93) ranked accessing CGM data in the top 3 priorities (20% [19/93] ranked highest, 9% [8/93] ranked second, and 14% [13/93] ranked third). This was followed by insulin pump data (39% [36/93] ranked in the top 3), communication with the diabetes team between visits (37% [34/93] ranked in the top 3), and laboratory test results (37% [34/93] ranked in the top 3) as useful features. Other features frequently ranked as highly useful included accessing diabetes care plans developed with the diabetes team and the ability to share diabetes care plans with other HCPs (Figure 2).

#### Diabetes HCPs

**Figure 3:**
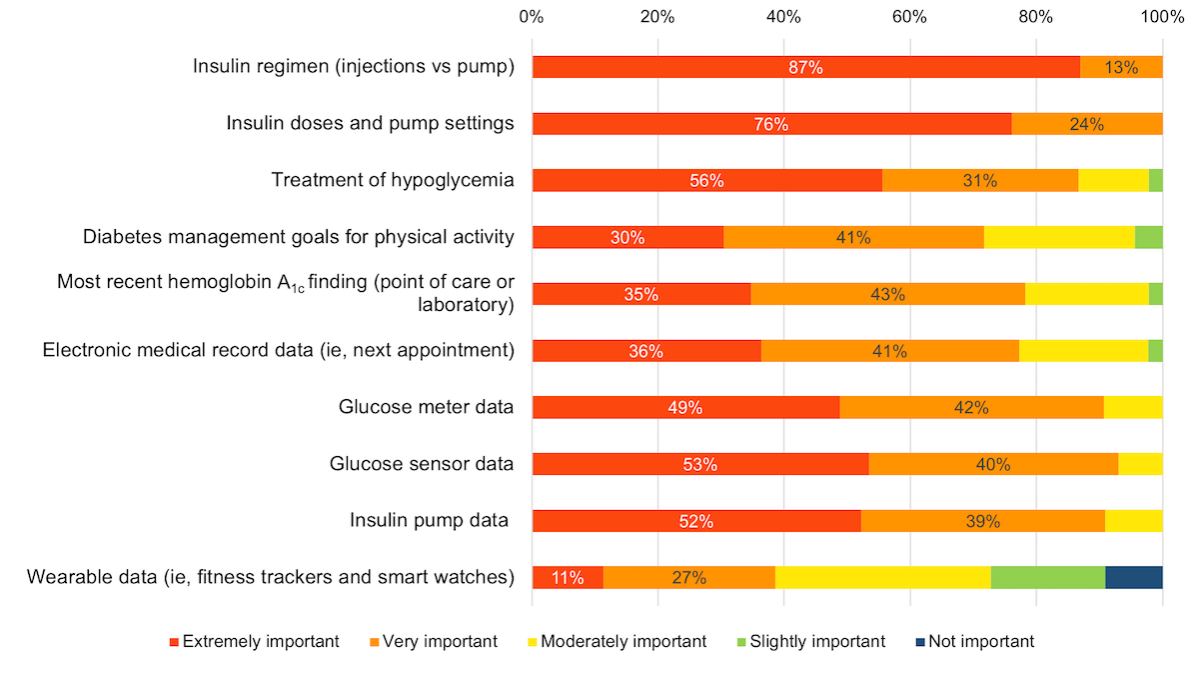
Importance of information that health care providers want to include in this digital platform. (“How important is it to include/integrate the following features/data in an integrated patient platform for T1D? *Select one answer per row*.”).

HCPs reported that in a digital single-point-of-access platform, it would be extremely important to include information about patient demographics (43/46, 94%), other medical diagnoses (41/46, 90%), non–diabetes-related medications (33/46, 72%), duration of diabetes (31/46, 68%), and age at diabetes diagnosis (29/46, 63%). HCPs also outlined the importance of including information on a patient’s insulin regimen, insulin doses, and glucometer and CGM data (Figure 3).

Further, HCPs stated it would be extremely important to include information on the results of diabetes complications screening laboratory tests (38/43, 88%), automated notifications for HCPs to complete complications screening based on Diabetes Canada Clinical Practice guidelines (33/44, 75%) [18], and patient anthropometric measurements such as blood pressure, height, and weight (31/43, 72%). Another essential feature for HCPs was integrating this digital platform with electronic medical records (EMRs).

When asked about securely communicating with patients and families through the platform, HCPs stated it would be very important to have a direct messaging feature (30/45, 67%), a platform email address (38/45, 84%), a link for videoconferencing (38/45, 84%), as well as the capability to manage clinic appointments (26/45, 58%).

### Perceptions About the Digital Platform and Likelihood of Use

#### Caregivers of Children and Youths Living With T1D

Caregivers reported that they would be likely to use the platform (65/98, 66%) and stated that the platform would be helpful for their child (83/98, 85%), themselves (97/99, 98%), as well as their HCPs (99/99, 100%) and researchers (99/99, 100%). Caregivers were very willing to share their child’s data with their diabetes health care team (97/97, 100%) and other HCPs (96/97, 99%). Still, they would be willing to only share some of their data with members outside the diabetes team, such as other family members (52/97, 54%), school health nurses (66/97, 68%), teachers (65/97, 67%) and after-school program staff (57/97, 59%).

#### Diabetes HCPs

HCPs stated that the integrated patient platform would be very helpful (38/44, 86%) and greatly simplify their care for children and youths living with T1D (21/45, 47%). A total of 75% (34/45) of HCPs were likely to use this platform. However, some (10/45, 22%) were undecided due to concerns such as integration with their current EMR, the need for additional documentation and duplication in charting, and that patient participation and engagement were necessary. HCPs also raised concerns related to inequitable access based on socioeconomic status and education, as well as challenges for non–English-speaking families or those who experience barriers in accessing technology or the internet.

## Discussion

### Overview

In this study, we found that caregivers of children and youths living with T1D and pediatric diabetes HCPs reported similar challenges in managing T1D as well as desirable features for a digital platform to address these challenges, such as access to data from diabetes devices, laboratory results, mental health support, and resources and tools to support diabetes self-management (including school support). For caregivers, the ability to communicate more easily and frequently with their diabetes care team, particularly between clinic visits, and share their diabetes care plans with others in their circle of care were also emphasized as critical features. Most HCPs and caregivers stated they were likely to use the described platform and that an integrative platform would be helpful for collaborative patient care. The results of our study confirm that a digital platform for collaborative use by both caregivers and HCPs (with some user-specific features) has the potential to address key pain points and ultimately improve the care experience.

Most diabetes health apps only seem to be designed for patients [3] and integrating preferences in the design of the diabetes digital apps from both patients and HCPs is limited [3,9-11,14,19]. To successfully implement digital solutions in health care, it is essential to involve end users in the design and development as early as possible. Our study is novel in addressing the needs of caregivers of children and youths living with T1D and pediatric HCPs, which includes physicians, dieticians, and diabetes nurses, at the conceptual stage in the user-centered design of the *TrustSphere Consortium’s* digital platform, which will increase its likelihood of meeting end users’ needs. Unique to our study, we found that caregivers would also want to share their child’s diabetes care plan not just with HCPs, but also with others caring for their children. Further, HCPs stated that they would also like to see diabetes complications screening laboratory tests as a feature on this digital platform, which has not been reported before.

Adult patients also desire mental health apps geared toward social support and well-being to be a part of diabetes digital apps [9,19]. In this study, insufficient access to mental health support was identified as one of the key challenges by caregivers and patients cared for by HCPs. Caregivers stated it’d be helpful to have links to peer group and social support networks as a feature on the digital platform (data not shown); however, this was not identified as one of the top 3 key features desired. While addressing unmet mental health needs is clearly a priority in this population, this remains a challenging service to provide safely and effectively through a mobile app. Attempting to meet this widespread need through a virtual provision of support would be outside the scope of the platform being designed as part of this study; however, the data on the current unmet need for mental health support will inform future strategic planning around partner services, resources, information, and peer support apps and links that may be integrated into the platform in future iterations.

Patients’ demographic characteristics play a role in the uptake of diabetes digital health apps. A recent systematic review revealed that younger, female adult patients, those with a higher level of education and higher monthly incomes are more likely to adopt digital health apps [14,19]. Similarly in our study, 76% (71/94) of the caregivers were female, 65% (59/91) had a college or university degree, and 74% (63/85) had an annual income bracket greater than CAD $100,000 (US $ 76,645.50; Table 1).

Apps that offer multiple features, including documentation, reminders, educational resources, consolidation of diabetes data from different diabetes devices, and nutritional support are more likely to foster long term patient engagement and use [14]. Our study results have uncovered key features as noted in Figures 2 and 3 that will guide the design of the integration of various diabetes data devices within our digital platform. Further, our team has adopted an existing digital health platform, Careteam Technologies, a *TrustSphere Consortium* industry partner, that will support health care coordination and collaboration among patients, their families, and HCPs. This will be done through a dashboard that will contain diabetes care plans (eg, recommendations of insulin doses), real-time data from connected devices, educational resources, and assigned tasks (eg, completion of blood work) to enhance personalization of pediatric diabetes care. Future developments of our app can look into integration with Learn Diabetes [20], a web-based educational platform developed at BCCH for children living with T1D. It has been shown that the successful implementation of innovative medical technologies depends on acceptance by all health care team members; therefore, it is essential to identify and address barriers to adopting a new digital platform in the clinical workflow as early as possible [21]. A study investigating barriers to uptake digital health technology in cardiovascular care identified poor internet connection and difficult-to-use technology as reported by patients and increased workload and perceived app usefulness as reported by HCPs [22]. Facilitators to improve the uptake of digital health technology included improved communication with clinicians, personalized components among patients and improved efficiency and organizational support among HCPs [22]. HCPs in our study identified key barriers that have been considered in the design and development of TrustSphere’s digital platform. For example, the platform must be synergistic with, not duplicative of, EMRs. Further, the onboarding process and workflow must support engagement from all HCPs, as well as caregivers, for the platform to succeed.

### Strengths and Limitations

Our study involved pediatric HCPs and caregivers of children and youths living with T1D as early as possible in the design and development of a digital integrative platform to optimize its potential to address key pain points, ensure ongoing usability, acceptance and adoption by HCPs and patients, and ultimately improve patient experience and outcomes.

User-centered design and digital app use are prone to selection bias since participants are often Caucasian, female, more educated, tech-savvy, and identify English as their primary language [19,23]. In addition, currently many diabetes management apps are unavailable in languages other than English [4]. This study is limited because caregivers of children and youths living with T1D were only recruited from a tertiary-level pediatric diabetes clinic at BCCH, which provides care to about 40%-50% of children and families living with diabetes in the province of BC. Also, the participants were mostly female and had a higher education and household annual income. We did not collect participant data on race or ethnicity, hence, making it difficult to generalize our findings to more diverse populations. A total of 25% of American adults with household incomes less than CAD $30,000 (US $22,791.85) do not own a smartphone. Of those who do, 27% are smartphone-only internet users, indicating they rely solely on their phone’s cellular data services [24]. The surveys were sent electronically to HCPs and caregivers in this study, so only those with access to a smartphone or the internet could easily participate. It is important to consider critical issues of equity and access so that new technology closes, rather than widens, the current divide in diabetes technology use across socioeconomic and educational levels [25].

The surveys in this study were distributed at 1 point in time to both caregivers and HCPs, to gather their perspectives on the current challenges and key features desired for this digital platform. As part of the user-centered design approach, following the quantitative data gathered through these needs assessment surveys, focus groups and web-based bulletin boards were conducted to collect qualitative data. This will inform the first stage of cocreating a digital platform for children living with T1D. The resulting prototype will be iteratively presented to caregivers of children and youths living with T1D and pediatric diabetes HCPs across BC in multiple rounds of usability testing (data forthcoming) with the integration of feedback and findings into a minimal viable product that will subsequently be tested in a clinical pilot study.

Future strategies to minimize the divide in the uptake of digital technology include automating and standardizing the collection of race, ethnicity, and primary language data, and obtaining input from ethnic minorities and those with limited English proficiency or poor digital literacy early in the usability testing process. Also, improving literacy can involve distributing educational materials in languages other than English, so patients and their families can better understand and interpret the information provided by digital apps [26]. It is challenging to develop policies that allow for equitable access to all forms of technology, such as smartphones or improving cellular data coverage or home internet access in rural areas; however, this can be argued if it relates to digital health technology. The UK National Health Service has extended the coverage of real-time CGM to all children living with T1D, including those from lower socioeconomic groups or ethnic minorities [27]. Similarly, BC PharmaCare, which covers medical devices and prescriptions, has expanded the approval of Dexcom G6 real-time CGM to all patients on intensive insulin therapy who are older than 2 years [28]. Policies such as these will be critical in minimizing and eliminating the digital divide and the associated disparities in health outcomes, and potentially expand the use and usefulness of an integrative T1D digital platform such as the one described here to more diverse populations.

### Conclusions

This study demonstrates the importance of implementing user-centered design as early as possible in developing new digital solutions to ensure optimal use, acceptance, and adoption by HCPs and patients alike. It is novel in identifying the needs of both caregivers of children and youths living with T1D and pediatric HCPs to inform the design of an integrative T1D platform. This study will also inform future digital innovation in the T1D space with an ongoing opportunity to use participatory, user-centered design to achieve higher-quality integrated digital health apps.

## Appendix

Multimedia Appendix 1 Survey Items used for Caregivers of Children & Youths Living with T1D.

Multimedia Appendix 2 Survey Items used for Pediatric Diabetes Healthcare Professionals.

## Abbreviations

BC: British Columbia
BCCH: BC Children’s Hospital
CGM: continuous glucose monitor
EMR: electronic medical record
HCP: health care provider
T1D: type 1 diabetes
