# Supplementary material for: Designing a Collaborative Patient-Centered Digital Health Platform for Pediatric Diabetes Care in British Columbia: Formative Needs Assessment by Caregivers of Children and Youths Living With Type 1 Diabetes and Health Care Providers
Source: JMIR Pediatr Parent. 2023 Jul 13;6:e46432. doi: 10.2196/46432 (PMC10375277; doi:10.2196/46432)
Supplement: Multimedia Appendix 1 [file pediatrics_v6i1e46432_app1.docx]

**Appendix 1:**

Survey Items used for Caregivers of Children & Youth Living with T1D

1. How old was your child when they were first diagnosed with T1D?

**[DROP DOWN RANGE 0-18]**

2. How old is your child now?

**[DROP DOWN RANGE 0-18]**

**[IF HAVE CHILD AGE 8+]**

3. Which of the following best describes the role **your child** plays when it comes to making decisions about their health?

*Please select one.*

They have primary decision-making responsibility

They share the decision-making responsibility

They have no role or influence in making decisions

4.  Do you or your child wear/use any of the following types of health monitoring equipment?

*Select all that apply.*

**[ROWS - RANDOMIZE]**Smart watches (e.g. Fitbit, Apple Watch)

Blood pressure monitoring

Continuous glucose monitoring system (CGMS)

Insulin Pump

Smart insulin pen

Health and wellbeing monitoring apps on smartphone

Do not use any of these **[EXCLUSIVE]**

Other (please specify)

**[COLUMNS]**

      Myself

      My child

5. What are the biggest challenges that you currently face related to caring for your child with T1D?

*Select your top 3.*

Access to a pediatric diabetes doctor

Access to a diabetes nurse educator

Access to a registered dietitian with experience in pediatric diabetes

Access to mental health support (social worker or psychologist)

Access to diabetes management technologies (i.e. insulin pumps, glucose sensors)

Support for your child’s diabetes care in school

Accessing your child’s medical information (i.e. glucose sensor data, pump data, lab test results)

Connecting with your diabetes team between visits

Other [SPECIFY]

6. Currently, how much of a challenge is it for you to keep track of all of your child’s health information?

*Select one.*

Never a challenge

Rarely a challenge

Sometimes a challenge

Usually a challenge

Always a challenge

7. Currently, how much of a challenge is it for you to share your child’s health information (including their care plan) between different healthcare providers who see your child?

*Select one.*

Never a challenge

Rarely a challenge

Sometimes a challenge

Usually a challenge

Always a challenge

8. How do you currently manage or keep track of all of your child’s health information?

*Select one.*

All paper copies (i.e. a binder or folder)

A mix of paper copies and digital (i.e. online/app/electronic) sources or copies

All digital sources or copies

Other [SPECIFY]

For the next set of questions, we’d like you to review the description of a potential digital platform (for example, website or app) that would connect health care providers, and allow them and you to access your child’s health information.

*A secure online platform that will be customized for child and youth patients and their caregivers, and will integrate a patient’s health information such as diagnoses, medications and treatments, appointments, lab test results, wearable data (e.g. FitBit), etc. This platform would use secure and trusted digital identification, and follow the highest healthcare industry and public standards of privacy protection. The platform would help make it easier for children and families to access their health information and care plans, and to communicate directly with healthcare providers. It would also allow users to share their health information and care plans, if desired, with others involved in their child’s care, as well as donate their data confidentially for research.*

9. How helpful do you think an integrated platform as described above would be to/for the following groups and/or individuals?

*Select one answer per row.*

**ROWS**

Your child

You as a parent/guardian

Doctors and other healthcare practitioners

Researchers

**COLUMNS**

Not at all helpful

Not very helpful

Somewhat helpful

Very helpful

Extremely helpful

10. If this platform was customized for children and youth with Type 1 Diabetes and their parents/caregivers, what integrated data or features would be most useful for you?

*Rank in priority.*

Continuous glucose monitoring system (CGMS) data

Glucometer data

Insulin pump data

Smartwatch /FitBit data

Lab test results

Access to searchable diabetes education resources

Link to a peer/social support network

Ability to manage appointment scheduling

Diabetes care plans developed with your diabetes team

Communication with your diabetes team between visits

Ability to share data and/or care plan with other healthcare providers

Ability to share data and/or care plan with others in your community who look after your child

11. If you could control what data or information is being shared through this platform, who else in your child’s care team would you want to share data or information with?

*Select one answer per row.*

**ROWS**

Diabetes healthcare team

Other healthcare providers

School health nurse

Teacher

Sports coach/after-school program staff

Other family members

Other (please specify)

**COLUMNS**

Share no data

Share some data

Share all data

12. What is the likelihood that you would use a platform like this?

*Select one.*

Very unlikely

Unlikely

Undecided

Likely

Very likely

The last few questions are for classification purposes only.

13. In what year were you born?

**[DROP DOWN RANGE 2004 to 1940]**

Prefer not to say

14. Do you have any of the following types of health care coverage?

*Choose all that apply.*

      Private health care coverage or insurance that my employer pays for

      Private health care coverage or insurance that I pay for

      No additional health care coverage **[EXCLUSIVE]**

15. Which of the following genders do you identify as?

*Please select one.*

Male

Female

Other

Prefer not to answer

16. Which of the following best describes the area you live?

*Please select one.*

Urban

      Suburban

      Rural

      Don’t know/prefer not to answer

17. Which of the following ranges best describes your **total annual household income** before taxes?

*Please select one.*

Less than $45,000

$45,000 to less than $75,000

$75,000 to less than $100,000

$100,000 to $150,000

$150,000 to $300,000

More than $300,000

Prefer not to answer

18. What is the highest level of education you have completed?

*Choose one.*

High school graduation or less

Some trade school

Graduated from trade school

Some college / university

University or college undergraduate degree, such as a bachelor’s degree

University or college graduate degree, such as a master’s or PhD

Prefer not to answer
