# Supplementary material for: Designing a Collaborative Patient-Centered Digital Health Platform for Pediatric Diabetes Care in British Columbia: Formative Needs Assessment by Caregivers of Children and Youths Living With Type 1 Diabetes and Health Care Providers
Source: JMIR Pediatr Parent. 2023 Jul 13;6:e46432. doi: 10.2196/46432 (PMC10375277; doi:10.2196/46432)
Supplement: Multimedia Appendix 2 [file pediatrics_v6i1e46432_app2.docx]

**Appendix 2:**

Survey Items used for Pediatric Diabetes Healthcare Professionals:

**A. Demographics & Current Practice:**

1. Which of the following best describes your role in the care of children and youth with Type 1 diabetes (T1D)?

*Select one.*

   Pediatrician

   Pediatric endocrinologist

   Family doctor

   Dietitian

   Diabetes nurse educator

   Social worker

         Other [Specify]

2. Where do you practice? (If you practice in more than one location, please choose the location in which the majority of your practice takes place)

*Select one.*

Fraser Health Authority

Interior Health Authority

Northern Health Authority

Vancouver Island Health Authority

Provincial Health Services Authority

Vancouver Coastal Health Authority

3. Using your best estimate, how many children and youth <18 years of age with T1D do you follow?

*Select one.*

   <25

   25 to <50

   50 to <100

   100 to <150

   150 to <200

   >200

4. Do you provide care to children and youth <18 years of age with T1D with a multi-disciplinary team (i.e. diabetes nurse educator and/or dietitian)?

*Select one.*

   Yes always

   Yes sometimes - at least 50% of the time

   Yes sometimes - less than 50% of the time

   No

   Not sure

5. What are the current key challenges that your T1D patients and their families report facing?

*Select top 3.*

Access to a pediatric diabetes doctor

Access to a diabetes nurse educator

Access to a registered dietitian with experience in pediatric diabetes

Access to mental health support (social worker or psychologist)

Access to diabetes management technologies (i.e. insulin pumps, glucose sensors)

Support for their child’s diabetes care in school

Accessing their child’s clinical information (i.e. glucose sensor data, pump data, lab investigations)

Connecting with their diabetes team between visits

Other [Specify]

6. When providing care to children and youth <18 years of age with T1D in your clinic, to what degree do you find each of the following to be a current challenge in your practice?

*Select one answer per row.*

**[ROWS]**  Seeing patients in diabetes clinic as frequently as I would like

   Accessing blood glucose meter data (i.e. logbooks) for a clinic visit

   Accessing glucose sensor uploads for a clinic visit

   Accessing insulin pump uploads for a clinic visit

   Accessing lab results for a clinic visit

   Communicating with patients and families between clinic visits

   Helping patients navigate technology (i.e. uploading pumps and glucose sensors)

   Knowing when to screen for complications and co-morbidities

   Motivating my patients to get their screening bloodwork done

   Accessing mental health support for patients and families

   Having sufficient human resources in my diabetes clinic

   Being able to create optimal care plans for patients

   Sharing and/or updating care plans with other members of a patient’s healthcare team

   Other [Specify]

**[COLUMNS]**

   Not a challenge

   Sometimes a challenge

   A frequent and/or major challenge

We are planning to develop a patient-centered integrated digital platform customized for patients and caregivers of children and youth with T1D, and for the healthcare providers who serve them. This integrated patient platform will use secure and trusted digital identification and be in compliance with the highest healthcare industry and public standards of privacy protection.  It will provide a single-point-of-access dashboard for care providers and patients that will integrate patient data from a range of sources including clinical data from EMRs and labs, glucose sensors and insulin pumps, and wearables (e.g. FitBit), and will enable the creation of personalized patient care plans. This integrated platform will allow for direct communication between patients/caregivers and members of their care team, and the ability for patients to share their medical data and diabetes care plans, if desired, with others individuals within their circle of care (i.e. primary care, nursing support services, etc).

In each of the categories below, please rate how important you believe it is to include each of the features listed in the Careteam for T1D integrated patient platform.

**B.**  **Patient Demographics:**

7. **How important** is it to include the following information about the patient in the integrated patient platform?

*Select one answer per row.*

**[ROWS]**Patient demographic information (i.e. name, age, sex, date of birth, health card number, etc.)

   Patient’s contact information (i.e. home address, phone number, email, etc.)

   Age at diabetes diagnosis

   How long the patient has had diabetes

   A list of the patient’s other medical diagnoses

   A list of the patient’s other health care providers

   A list of the patient’s other non-diabetes related medications

**[COLUMNS]**

   Not important

   Slightly important

   Moderately important

   Very important

   Extremely important

8. Is there any other patient demographic information not listed above that you feel would be important to include? **[Open Text]**

**C. Diabetes Care Plan:**

The diabetes care plan within the integrated patient platform will include a patient’s personalized self-management plan and will support collaborative goal setting between the patient/family and the healthcare provider. With the appropriate permissions, the care plan can be modified by the patient/family and/or a health care provider. The patient/family can share the care plan with others within their circle of care (i.e. family doctor, pediatrician, local diabetes nurse educator, nursing support services, schoolteacher, babysitter, etc).

9.  **How important** is it to include the following features in a personalized diabetes care plan within the integrated patient platform?

*Select one answer per row.*

**[ROWS]**  Insulin Regimen (i.e. MDI, TID injections, pump therapy)

Summary of the patient’s age-specific glucose and A1C targets as defined by Diabetes Canada Clinical Practice Guidelines

Insulin doses and/or settings (i.e. sliding scales, basal insulin doses, insulin to carbohydrate ratio, sensitivity factor, Basal rates)

Personalized patient goals (i.e. A1C goal; frequency of daily blood glucose monitoring; % time in range goal)

   Treatment of hypoglycemia

   Diabetes management goals for physical activity

   Most recent A1C result (point of care or laboratory)

   A1C results over the last 12 months (point of care or laboratory)

**[COLUMNS]**

   Not important

   Slightly important

   Moderately important

   Very important

   Extremely important

10. Are there any other features in a diabetes care plan not listed above that you feel would be important to include? **[Open Text]**

**D. Diabetes Complications Screening:**

11. **How important** is it to include the following in the integrated patient platform to facilitate complications screening?

*Select one answer per row.*

**[ROWS]**

Notifications to conduct complications screening based on Diabetes Canada Clinical Practice Guidelines for pediatric T1D

Patient measurements including most recent blood pressure, height, weight and body mass index

Results of diabetes complications screening laboratory tests (from provincial labs and regardless of ordering clinician)

         A printable pre-filled and signed laboratory requisition for patients

**[COLUMNS]**

   Not important

   Slightly important

   Moderately important

   Very important

   Extremely important

**E. Integration of Patient Data:**

12. **How important** is it to integrate the following data (from other sources) into the integrated patient platform?

*Select one answer per row.*

**[ROWS]**

         Electronic Medical Record data (i.e. appointment days/times, consultation notes)

         Glucose meter data

         Continuous or Flash glucose monitoring data

         Insulin pump upload data

         Wearable data (i.e. fitness trackers, smart watches)

         BC Pharmanet data

**[COLUMNS]**

   Not important

   Slightly important

   Moderately important

   Very important

   Extremely important

13. Are there any other sources of external patient data not listed above that you feel would be important to integrate? **[Open Text]**

**F.  Communicating with your Patients**

14.  **How important** is it to have the following options for securely communicating with patients and/or their families via the integrated patient platform?

*Select one answer per row.*

**[ROWS]**

   Direct messaging

   Email

   Video conference (i.e. zoom for health)

**[COLUMNS]**

   Not important

   Slightly important

   Moderately important

   Very important

   Extremely important

15. **How important** is it to be able to manage appointments (scheduling, re-scheduling, etc) with patients via the integrated patient platform?

*Select one.*

**[COLUMNS]**

   Not important

   Slightly important

   Moderately important

   Very important

   Extremely important

16. In your diabetes clinic, what methods of communication do you use with patients and/or their parents/guardians?

*Select all that apply.*

**[ROWS]**

Telephone

Email

Text message/SMS

Other [Specify]

17. In your diabetes clinic, who is **primarily** responsible for checking and responding to messages about scheduling from patients and/or their parents/guardians?

*Select one.*

**[ROWS]**

Physician

Dietician

Diabetes Nurse Educator

Social Worker

Administrative Assistant

Other [Specify]

18. In your diabetes clinic, who is **primarily** responsible for checking and responding to messages about non-urgent diabetes management questions from patients and/or their parents/guardians?

*Select one.*

**[ROWS]**

Physician

Dietician

Diabetes Nurse Educator

Social Worker

Administrative Assistant

Other [Specify]

19. In your diabetes clinic, who is **primarily** responsible for checking and responding to messages about urgent diabetes management questions from patients and/or their parents/guardians?

*Select one.*

**[ROWS]**

Physician

Dietician

Diabetes Nurse Educator

Social Worker

Administrative Assistant

Other [Specify]

**G. Platform Use:**

20. How helpful do you think the integrated patient platform as described above would be in helping you care for children and youth with T1D?

*Select one.*

**[COLUMNS]**

   Not helpful

   Slightly helpful

   Moderately helpful

   Very helpful

   Extremely helpful

21. Do you think that having a new digital platform as described above for use by your patients and in your clinic would simplify or complicate your care of children and youth with T1D?

*Select one.*

**[COLUMNS]**

   Would greatly simplify

   Would slightly simplify

   Would slightly complicate

   Would greatly complicate

22.  What is the likelihood that you would use the integrated patient platform when caring for children and youth with Type 1 Diabetes?

*Select one.*

**[COLUMNS]**

   Very unlikely [>Branching to 24B if selected]

   Unlikely [>Branching to 24B if selected]

   Undecided [>Branching to 24C if selected]

   Likely

   Very likely

  22B. Please indicate why you might not use the integrated patient platform.

*Select all that apply.*

**[ROWS]**

         I don’t think an integrated platform will benefit my patients

            I don’t want to learn how to use a new technological platform

   I don’t have time to use a patient integrated platform in my practice

I don’t think my patients will use a patient integrated platform

I am concerned about technology burnout for patients/caregivers

I am concerned about technology burnout for healthcare providers

I don’t trust that my patients’ information will be protected securely

Other [Specify]

22C. Is there a particular barrier to use that needs to be addressed, or an additional functionality that needs to be included, in order for you to be more likely to consider using the platform with your patients? **[Open Text]**

23. Do you have any other comments or suggestions that you would like to share related to the development of a patient-centered integrated platform for children and youth living with T1D?

**[Open Text]**
